# Reducing transmission in multiple settings is required to eliminate the risk of major Ebola outbreaks: a mathematical modelling study

**DOI:** 10.1098/rsif.2024.0765

**Published:** 2025-03-19

**Authors:** Abbie Evans, William Hart, Stefano Longobardi, Rajat Desikan, Anna Sher, Robin Thompson

**Affiliations:** ^1^ Mathematical Institute, University of Oxford, Oxford, UK; ^2^ Non-Clinical Safety, Research Technologies, GSK, Stevenage, UK; ^3^ Clinical Pharmacology Modelling and Simulation, Development, GSK, Stevenage, UK; ^4^ Clinical Pharmacology Modelling and Simulation, Development, GSK, MA, USA

**Keywords:** Ebola virus disease, infectious disease outbreak, non-pharmaceutical interventions, branching process, probability of a major outbreak

## Abstract

The Ebola virus (EV) persists in animal populations, with zoonotic transmission to humans occurring every few months or years. When zoonotic transmission arises, it is important to understand which interventions are most effective at preventing a major outbreak driven by human-to-human transmission. Here, we analyse a mathematical model of EV transmission and calculate the probability of a major outbreak starting from a single introduced case. We consider community, funeral and healthcare facility transmission and conduct sensitivity analyses to explore the effects of non-pharmaceutical interventions (NPIs) that influence these transmission routes. We find that, if the index case is treated in the community, then the elimination of transmission at funerals reduces the probability of a major outbreak substantially (from 0.410 to 0.066 under our baseline model parametrization). However, eliminating the risk of major outbreaks entirely requires combinations of measures that limit transmission in different settings, such as community engagement to promote safe burial practices and implementation of barrier nursing in healthcare facilities. In addition to generating insights into the drivers of Ebola outbreaks, this research provides a modelling framework for assessing the effectiveness of interventions at mitigating outbreaks of other infectious diseases with transmission in multiple settings.

## 1. Introduction

In the early stages of an emerging infectious disease outbreak, a key public health policy question is whether initial cases are likely to lead to a major outbreak or whether the outbreak will fade out instead [1–4]. Relatedly, policy advisors seek to understand which interventions can be introduced to reduce the impact of the outbreak on the host population, including considering whether outbreak elimination is a realistic possibility [5–7].

Mathematical models have been used to guide policy-making during outbreaks of a range of diseases, including estimation of the so-called ‘probability of a major outbreak’ (i.e. the probability that early cases will initiate a large outbreak, as opposed to the outbreak fading out with few cases) [3,8–11]. One way to estimate this quantity is through repeated simulation of a stochastic epidemic model. Starting from a small number of cases, the probability of a major outbreak may be defined to be the proportion of simulated outbreaks in which the total number of cases exceeds a specified threshold [3,8]. However, since repeated model simulation can require substantial computational resources, an alternative approach is instead to estimate the probability of a major outbreak analytically (or numerically) using branching process models [8]. In that case, the probability of a major outbreak is typically defined to be the probability that the branching process does not become extinct [8,12].

Branching process models are used frequently in epidemiological modelling studies. For example, Kelly *et al*. [13] deployed a branching process model during the 2018 Ebola outbreak in Équateur Province, Democratic Republic of the Congo, to project the size and duration of that outbreak in real time, including considering how those quantities would vary with different vaccination coverage levels. Toth *et al.* [4] used a similar model in the context of the 2014−2016 Ebola epidemic in West Africa to explore the impact of the timing of the introduction of control interventions on the risk posed by Ebola outbreaks, including considering different levels of heterogeneity in transmission between infected individuals. As noted above, an important use of branching process models is to estimate the probability of a major outbreak, which has been undertaken in the context of diseases such as COVID-19 [9,14] and influenza [15], as well as Ebola [5,16–18]. Substantial epidemiological complexity has been integrated into branching process models, including age structure and the potential for asymptomatic transmission [19,20]. Calculations of the probability of a major outbreak have been adapted to account for heterogeneity in infectiousness between hosts [21], seasonally varying environments [22,23], spatial structure [24] and multiple pathogen strains [25].

Here, we adapt and extend a previous branching process model of Ebola virus (EV) transmission [26] that was used to analyse health system demand in Liberia during the 2014−2016 Ebola epidemic. Rather than using the model to explore transmission scenarios once a large outbreak is ongoing (as in [26]), a novel aspect of the present study is that we focus on the earliest stages of an Ebola outbreak and derive equations describing the probability of a major outbreak under this model analytically. The EV can be transmitted in different settings, in addition to regular community transmission. For example, in the 2014−2016 Ebola epidemic in West Africa, unsafe burial practices occurred due to cultural beliefs within host populations [27], with risky behaviours such as washing and touching corpses [28,29] leading to a high risk of post-death transmission. In addition, substantial EV transmission can occur in healthcare settings, particularly when infection control precautions are not followed strictly [30]. These different transmission routes are reflected in the transmission model used in this study, and we conduct sensitivity analyses to identify the transmission routes that, if limited, would lead to the largest reductions in the probability of a major outbreak.

Through our sensitivity analyses, we find that unsafe burials at funerals contribute substantially to the probability of a major outbreak. This indicates that increased compliance with safe burial practices would reduce outbreak risks. However, under the assumed transmission model, even complete compliance with safe burial practices does not eliminate the probability of a major outbreak entirely, since community transmission and transmission in healthcare facilities can drive outbreaks. We, therefore, consider limiting different transmission routes in combination, demonstrating that intervention strategies that affect multiple transmission routes must be adopted and compliance must be high for the probability of a major outbreak to be reduced to zero. This highlights the substantial challenge in preventing Ebola outbreaks, which requires mitigation of transmission in multiple settings.

## 2. Methods

### 2.1. Transmission model

We consider a mathematical model of EV transmission that is based on the model by Drake *et al.* [26]. In our model, infections occur according to a branching process, and infected individuals are either treated in the community (C) or at a healthcare facility (H). We define $p_{d}$ to be the case fatality rate for individuals who are treated in the community and $p_{f}$ to be the probability that an individual who dies in the community then undergoes an unsafe burial. An overall proportion $p_{b}=p_{d}\times p_{f}$ of individuals who are treated in the community, therefore, die and undergo an unsafe burial. Those individuals transition to the F compartment of the model and pose an additional transmission risk.

**Figure 1.**
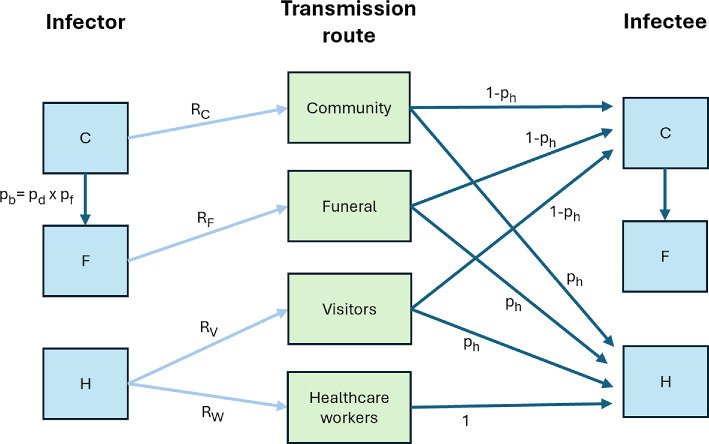
Schematic showing the different transmission routes in the branching process model. Infected individuals who are treated in the community (C) generate $R_{C}$ infections each on average. Additionally, a proportion $p_{b}$ of infected individuals who are treated in the community die and are buried unsafely (F), generating $R_{F}$ further infections each (on average). Cases who are treated in a healthcare facility (H) infect $R_{V}$ visitors to the facility and $R_{W}$ healthcare workers on average. Other than infected healthcare workers, who are treated in healthcare facilities, each new case has probability $p_{h}$ of being treated in a healthcare facility (thereby entering the H compartment; otherwise, the new infected individual enters the C compartment). In this figure, the ‘R’ variables representing expected numbers of new infections are shown with light blue arrows, and the ‘p’ variables denoting transition probabilities are shown with dark blue arrows.

A schematic illustrating the different transmission routes in the model is shown in figure 1. Individuals who are treated in the community generate $R_{C}$ secondary infections each on average, excluding infections at funerals. Individuals who die and are buried unsafely generate an additional $R_{F}$ infections each on average.

Individuals who are treated in healthcare facilities are assumed to generate new infections only following admission to those facilities. This assumption is based on the observation that infected individuals typically only become infectious after an initial period of dry symptoms during which they are not yet infectious [31]. Each infected individual who is admitted to a healthcare facility infects $R_{V}$ visitors to the facility and $R_{W}$ healthcare workers on average. Excluding infected healthcare workers (who are all treated in healthcare facilities), a proportion $p_{h}$ of new cases are treated in healthcare facilities with the remaining proportion $1-p_{h}$ being treated in the community.

While the parameters $R_{C}$, $R_{F}$, $R_{V}$ and $R_{W}$ represent average values, the numbers of infections arising via each transmission route are assumed to be drawn from geometric distributions (as is typically assumed when using compartmental epidemic models in which individuals spend an exponentially distributed period in each infectious compartment [8]).

### 2.2. Model parametrization

Data from previous Ebola virus disease (EVD) outbreaks were used to inform the values of the parameters of the transmission model (the baseline values used in our analyses are shown in table 1).

**Table 1.** Parameters of the model and the baseline values used in our analyses. Parameter values were obtained from the literature as described in the text.

Parameter	Definition	Baseline value	References
$p_{h}$	Probability of admission to a healthcare facility	0.6	[26]
$p_{d}$	Case fatality ratio for community cases	0.7	[26,32]
$p_{f}$	Probability of unsafe burial given death in the community	0.571	[26]
$R_{C}$	Expected number of infections generated by a community case (excluding funeral transmission)	1.04	[33]
$R_{F}$	Expected number of infections arising at an unsafe burial	5.9	[26]
$R_{V}$	Expected number of visitors infected by a case who is treated in a healthcare facility	0.25	[26]
$R_{W}$	Expected number of healthcare workers infected by a case who is treated in a healthcare facility	0.830	[26]

An outbreak investigation in the Democratic Republic of the Congo identified whether or not contacts of 27 primary community cases became infected [33]. In that study, 28 contacts experienced an illness that met the case definition. Consequently, we set the expected number of infections generated by each community case in our model to be $R_{C}=28/27$.

The proportion of community cases who die and undergo an unsafe burial, $p_{b}$, is given by the product of the case fatality ratio ($p_{d}$) and the probability that a community case who dies is buried unsafely ($p_{f}$). Based on studies from the 2014−2016 Ebola epidemic in West Africa [26,32], we set the case fatality ratio to be $p_{d}=0.7$, although we note that there is substantial uncertainty in this value with case fatality ratios lying in the range of 0.25−0.9 in previous Ebola outbreaks [30]. Of the community cases that end in fatality, $p_{f}=0.4/0.7$ are assumed to be buried unsafely. The value of $p_{f}$ was chosen so that the proportion of community cases who either recover or undergo a safe burial is $1-p_{b}=0.6$ (as in the study by Drake *et al.* [26]).

In healthcare facilities, there can be a high frequency of contact between cases and healthcare workers. We assumed that an individual who is treated in a healthcare facility has four times as many contacts, excluding visitor contacts, as an individual who is treated in the community (this is in line with the range of two to five used in the model by Drake *et al.* [26]). However, due to the increased awareness of infection risk in healthcare facilities, we assumed that the transmission risk is multiplied by a factor of 0.2 [26] so that the expected number of infections generated in healthcare workers by each case who is treated in a healthcare facility is $R_{W}=4\times 0.2\times R_{C}=0.830$.

The remaining parameter values used were taken directly from the study by Drake *et al*. [26] and are reported in table 1.

### 2.3. Probability of a major outbreak

To calculate the probability of a major outbreak starting from a single case introduced into the population, we follow the ‘first step analysis’ method outlined by Southall *et al*. [8]. Specifically, we derive a system of nonlinear simultaneous equations from which the probability of a major outbreak starting from a case who is treated in the community ($\pi_{C}$) and the probability of a major outbreak starting from a case who is treated in a healthcare facility ($\pi_{H}$) can be calculated.

We denote the probability of a major outbreak not occurring, starting from i cases who are treated in the community (C), j cases who are undergoing an unsafe burial (F) and k cases who are treated in a healthcare facility (H), by $q_{ijk}$. This notation is similar to that used in §4.1.2 of the article by Southall *et al*. [8] in which those authors also calculated the probability of a major outbreak not occurring starting from infectious individuals in different states of an epidemiological transmission model. Here, starting from a single case who is treated in the community, such that i=1, j=0 and k=0, there are four possibilities for the next event. The possible events, and the probabilities that each is the next event, are as follows:

— Infection of a new case who is treated in the community, with probability $\frac{\left(1-p_{h}\right)R_{C}}{R_{C}+1}$.
— Infection of a new case who is treated in a healthcare facility, with probability $\frac{p_{h}R_{C}}{R_{C}+1}$.
— The existing case dies and undergoes an unsafe burial, with probability $\frac{p_{b}}{R_{C}+1}$.
— The existing case recovers or dies and undergoes a safe burial, with probability $\frac{1-p_{b}}{R_{C}+1}$.

We note that these probabilities assume that the possible events occur according to competing exponentially distributed waiting times (which is consistent with the assumption that the case generates a geometrically distributed number of community transmissions). The numerator of each expression represents the occurrence rate of each specific event, and the denominator, $R_{C}+1$, is the total occurrence rate of all possible events (in time units such that any community case either dies or recovers at total rate one). Conditioning on which of these four events occurs next, and applying the law of total probability, gives


$$q_{100}=\frac{\left(1-p_{h}\right)R_{C}}{R_{C}+1}q_{200}+\frac{p_{h}R_{C}}{R_{C}+1}q_{101}+\frac{p_{b}}{R_{C}+1}q_{010}+\frac{1-p_{b}}{R_{C}+1}q_{000}.$$

Noting that a major outbreak will not occur if there are no cases gives $q_{000}=1$. Since infections are assumed to occur according to a branching process, chains of transmission arising from different cases are independent. Consequently, $q_{200}=q_{100}^{2}$ and $q_{101}=q_{100}q_{001}$, and so


(2.1) $$q_{100}=\frac{\left(1-p_{h}\right)R_{C}}{R_{C}+1}q_{100}^{2}+\frac{p_{h}R_{C}}{R_{C}+1}q_{100}q_{001}+\frac{p_{b}}{R_{C}+1}q_{010}+\frac{1-p_{b}}{R_{C}+1}.$$

Analogous equations can be derived for the probability of a major outbreak not occurring starting from either a single case who is undergoing an unsafe burial or a single case who is treated in a healthcare facility, leading to


(2.2) $$q_{010}=\frac{\left(1-p_{h}\right)R_{F}}{R_{F}+1}q_{100}q_{010}+\frac{p_{h}R_{F}}{R_{F}+1}q_{010}q_{001}+\frac{1}{R_{F}+1},$$

(2.3) $$q_{001}=\frac{\left(1-p_{h}\right)R_{V}}{R_{V}+R_{W}+1}q_{100}q_{001}+\frac{p_{h}R_{V}+R_{W}}{R_{V}+R_{W}+1}q_{001}^{2}+\frac{1}{R_{V}+R_{W}+1}.$$

The system of nonlinear simultaneous equations (2.1), (2.2) and (2.3) can be solved numerically to obtain $q_{100}$, $q_{010}$ and $q_{001}$ (specifically, we take the smallest non-negative solution, as described by Southall *et al.* [8]; we do this using the ‘root’ method from the scipy.optimise library in Python). Then, the probability of a major outbreak following the introduction of a single infected case who is treated in the community is given by $\pi_{C}=1-q_{100}$, and the probability of a major outbreak following the introduction of a single infected case who is treated in a healthcare facility is given by $\pi_{H}=1-q_{001}$.

### 2.4. Sensitivity analyses

To investigate the factors that contribute most substantially to the probability of a major outbreak, we undertake local and global sensitivity analyses. Specifically, we consider the dependence of $\pi_{C}$ and $\pi_{H}$ on a range of model parameters that could be affected by non-pharmaceutical interventions (NPIs; namely $R_{C}$, $p_{f}$, $R_{V}$, $R_{W}$ and $p_{h}$).

To conduct a local sensitivity analysis to explore the effect of the parameter $R_{C}$ on the probability of a major outbreak starting from a single case who is treated in the community, we calculate the sensitivity of $\pi_{C}$ to $R_{C}$ using the formula


$$\chi \left(\pi_{C},R_{C}\right)=R_{C}\left|\frac{\partial \pi_{C}}{\partial R_{C}}\right|,$$

evaluated at the baseline values of the model parameters (shown in table 1). Analogous expressions are used to calculate the sensitivity of either $\pi_{C}$ or $\pi_{H}$ to a range of different model parameters. We note that, in some studies (e.g. [34,35]), sensitivity indices such as $\chi \left(\pi_{C},R_{C}\right)$ have included a multiplicative factor of the reciprocal of the dependent variable (i.e. our expression for $\chi \left(\pi_{C},R_{C}\right)$ would be divided by the baseline value of $\pi_{C}$). However, since this is not required to compare the sensitivity of $\pi_{C}$ to different model parameters (each sensitivity index for $\pi_{C}$ would be divided by the same value), we omit that factor here.

Since our local sensitivity analyses only determine the sensitivity of $\pi_{C}$ and $\pi_{H}$ to small perturbations around our baseline parameter values, we also conduct global sensitivity analyses. To do this, we calculate first-order and total-order Sobol’ indices that attribute variations in $\pi_{C}$ and $\pi_{H}$ to individual model parameters ($R_{C}$, $p_{f}$, $R_{V}$, $R_{W}$ and $p_{h}$), using the SALib Python package [36,37]. The first-order Sobol’ index for a given parameter represents the direct effect that the parameter has on the specified output (i.e. $\pi_{C}$ or $\pi_{H}$). The total-order Sobol’ index reflects the effect that the parameter, and parameter combinations that include that parameter, have on the output. The difference between the total-order Sobol’ index and the first-order Sobol’ index for a given parameter therefore reflects the extent to which interactions between the parameter under consideration and other parameters affect $\pi_{C}$ or $\pi_{H}$.

By fixing the value of any specified model parameter and calculating the Sobol’ indices for the other parameters, the relative importance of each remaining model parameter at the specified value of the fixed parameter can be calculated (we use this approach to determine the relative importance of $R_{C}$, $p_{f}$, $R_{V}$ and $R_{W}$ in determining $\pi_{C}$ for populations with different levels of access to healthcare—i.e. different fixed values of $p_{h}$). Further details about first-order and total-order Sobol’ indices are included in the electronic supplementary material.

## 3. Results

### 3.1. Probability of a major outbreak

We began by calculating the probability of a major outbreak starting from a single case who is treated in the community ($\pi_{C}$) and the probability of a major outbreak starting from a single case who is treated in a healthcare facility ($\pi_{H}$) for our baseline model parameter values (table 1). This gave major outbreak probabilities of $\pi_{C}=0.410$ and $\pi_{H}=0.176$.

**Figure 2.**
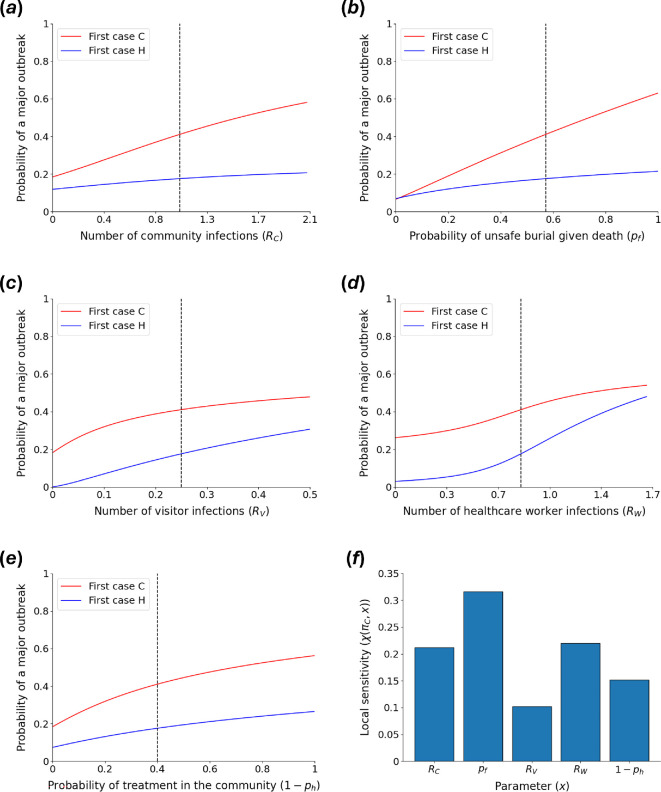
Effects of the values of different model parameters on the probability of a major outbreak. (*a*) The dependence of the probability of a major outbreak starting with either a case treated in the community ($\pi_{C}$—red) or a case treated in a healthcare facility ($\pi_{H}$—blue) on the expected number of community infections generated by each case who is treated in the community ($R_{C}$). The baseline value of $R_{C}$ is represented by the vertical black dashed line. All parameters other than $R_{C}$ are held fixed at their baseline values (table 1). (*b*) Analogous to *a*, but exploring the dependence of $\pi_{C}$ and $\pi_{H}$ on the probability that a community case who dies is buried unsafely ($p_{f}$). (*c*) Analogous to *a*, but exploring the dependence of $\pi_{C}$ and $\pi_{H}$ on the expected number of visitors who are infected by each case who is treated in a healthcare facility ($R_{V}$). (*d*) Analogous to *a*, but exploring the dependence of $\pi_{C}$ and $\pi_{H}$ on the expected number of healthcare workers who are infected by each case who is treated in a healthcare facility ($R_{W}$). (*e)* Analogous to *a*, but exploring the dependence of $\pi_{C}$ and $\pi_{H}$ on the probability that each case (excluding healthcare workers) is treated in the community ($1-p_{h}$). (*f*) Local sensitivity analysis to explore the dependence of $\pi_{C}$ on different model parameters (i.e. $\chi \left(\pi_{C},x\right)$, for $x=R_{C},p_{f},R_{V},R_{W}$ and $1-p_{h}$).

However, rather than seeking to calculate precise values of $\pi_{C}$ and $\pi_{H}$, our main goal was to identify the model parameters that affect the probability of a major outbreak most, to provide insights into NPIs that can reduce the risk that major outbreaks occur. In figure 2*a*–*e*, we plot $\pi_{C}$ and $\pi_{H}$ as functions of different model parameter values, with the remaining parameters fixed at their baseline values (for example, in figure 2*a*, the value of $R_{C}$ is varied while the values of all other parameters are fixed at the values shown in table 1). Notably, if the index case in the outbreak is treated in the community, then it is impossible to reduce $\pi_{C}$ to zero by changing the value of any individual model parameter. This suggests that any NPI that affects the value of only one model parameter will be unable to prevent major outbreaks entirely. On the other hand, if the index case is treated in a healthcare facility, $\pi_{H}$ is reduced to zero if transmission to visitors can be eliminated entirely (figure 2*c*).

We then conducted a local sensitivity analysis to identify the model parameters that, if altered, would lead to the fastest immediate reduction in $\pi_{C}$ from its baseline value (figure 2*f*). This analysis suggests that key parameters for reducing the probability of a major outbreak if the index case is treated in the community include $p_{f}$ (i.e. the probability that a case who dies in the community undergoes an unsafe burial), $R_{W}$ (i.e. the expected number of healthcare workers infected by each case who is treated in a healthcare facility) and $R_{C}$ (i.e. the expected number of transmissions by each case who is treated in the community, excluding funeral transmissions). Consequently, NPIs targeting these parameter values (e.g. public information campaigns to encourage safe burial practices and barrier nursing in healthcare facilities) might be expected to be particularly effective.

Since index cases in historical Ebola outbreaks have tended to be infected in rural areas [38] with limited access to healthcare, we contend that calculation of $\pi_{C}$ is particularly important. However, we note that if the index case is treated in a healthcare facility rather than in the community, then $R_{W}$ (and, to a lesser extent, $R_{V}$) becomes a particularly important factor (electronic supplementary material, figure S1). This further highlights the importance of measures to mitigate transmission in healthcare facilities to prevent major outbreaks.

In addition to conducting local sensitivity analyses, we undertook global sensitivity analyses using the SALib Python package to explore the dependence of $\pi_{C}$ on different model parameters both away from their baseline values and accounting for interactions with other parameters. From our analysis in electronic supplementary material, figure S2, we again found that $p_{f}$ (i.e. the probability that a case who dies in the community undergoes an unsafe burial) is a key parameter underlying the probability of a major outbreak.

The difference between electronic supplementary material, figures S2A and S2B, indicates that interactions between $p_{h}$ and other model parameters affect $\pi_{C}$ substantially. We therefore also conducted global sensitivity analyses for different fixed values of $p_{h}$, again using the SALib Python package (electronic supplementary material, figure S3). This showed that, in a population in which access to healthcare is limited (so that $p_{h}$ takes a low value), $p_{f}$ is once again a key driver of the value of $\pi_{C}$. However, in populations with substantial healthcare access (so that $p_{h}$ takes a high value), the parameter $R_{W}$ (i.e. the expected number of healthcare workers infected by a case who is treated in a healthcare facility) is instead a key determinant of $\pi_{C}$. This provides quantitative evidence to support the intuition that NPIs that reduce funeral transmission would be expected to be most effective in populations with limited healthcare access, and NPIs that limit transmission to healthcare workers would be expected to be most effective in populations with good healthcare access.

### 3.2. Using multiple interventions to reduce the probability of a major outbreak

**Figure 3.**
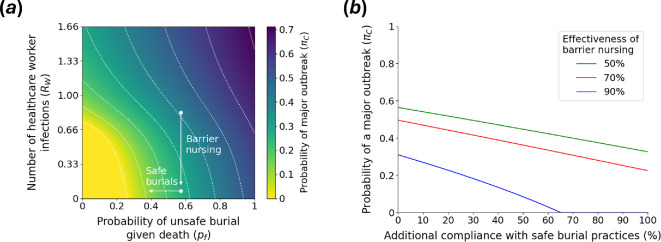
Reducing the probability of a major outbreak using combinations of interventions that affect transmission in multiple settings. (*a*) The effect of varying the probability of unsafe burial given death in the community ($p_{f}$) and the expected number of healthcare workers infected by each case who is treated in a healthcare facility ($R_{W}$) on the probability of a major outbreak starting from a single community case ($\pi_{C}$). White arrows are illustrative, indicating the combined effects of measures such as barrier nursing (reducing $R_{W}$) and adoption of safe burial practices (reducing $p_{f}$). White dotted lines represent contours along which $\pi_{C}$ takes fixed values. (*b*) The probability of a major outbreak starting from a single community case ($\pi_{C}$) as a function of additional compliance with safe burial practices. A value of 0% additional compliance corresponds to the baseline value of $p_{f}=0.571$, and a value of 100% additional compliance corresponds to $p_{f}=0$. Results are shown for different assumed values of the effectiveness of barrier nursing (i.e. different percentage reductions in $R_{W}$).

Since we found above that NPIs that affect only one model parameter are not able to eliminate the risk of major outbreaks entirely under the assumed transmission model and parameterization (figure 2*a*–*e*), we went on to explore whether varying multiple parameters in combination can reduce $\pi_{C}$ to zero. Since $p_{f}$ and $R_{W}$ are key parameters underlying $\pi_{C}$, we calculated $\pi_{C}$ for different combinations of values of these parameters (figure 3*a*). As shown in figure 3*a*, reducing $p_{f}$ and $R_{W}$ simultaneously has the potential to eliminate the risk of major outbreaks (with a sufficient reduction in $p_{f}$ and $R_{W}$; yellow region in the bottom left of figure 3*a*).

While NPIs targeting $p_{f}$ and $R_{W}$ may provide promising avenues for eliminating the risk of major Ebola outbreaks, we note that combinations of other parameters exist for which elimination of the probability of a major outbreak is impossible (electronic supplementary material, figure S4). Furthermore, high compliance with interventions affecting $p_{f}$ and $R_{W}$ would be required for the elimination of $\pi_{C}$ to be a realistic possibility. For example, if barrier nursing is assumed to reduce transmissions to healthcare workers by 90%, then alongside the implementation of barrier nursing, more than 65.1% of fatal cases in the community who would otherwise have been buried unsafely would need to be buried safely to reduce $\pi_{C}$ to zero (blue line in figure 3*b*). If barrier nursing only reduces 50% (green line in figure 3*b*) or 70% (red line in figure 3*b*) of transmissions to healthcare workers, then not even 100% compliance with safe burial practices (alongside barrier nursing) could eliminate $\pi_{C}$ completely. For reference, in the study by Drake *et al.* [26], the effectiveness of barrier nursing was estimated to be around 80%.

## 4. Discussion

Ebola outbreaks occur regularly in sub-Saharan Africa and are responsible for devastating consequences. Determining the public health measures that are most effective at reducing the risk that early human infections initiate a major outbreak is therefore a key challenge. In this article, we adapted a previously published Ebola outbreak model by Drake *et al.* [26] that distinguishes transmission at funerals, in healthcare settings and in the wider community (figure 1). We showed how the probability that an initial case (who is either treated in the community or in a healthcare facility) leads to a major outbreak can be calculated without requiring repeated model simulation. We considered the sensitivity of the probability of a major outbreak to the model’s parameters and demonstrated that key factors underlying the probability of a major outbreak include the proportion of fatal cases in the community that are buried unsafely and the extent of transmission to healthcare workers (figure 2). This suggests that NPIs that reduce transmission at funerals and in healthcare settings, such as community engagement to promote safe burial practices, and barrier nursing, might be expected to be particularly effective in terms of reducing the risk of large outbreaks.

In addition, we demonstrated that the most effective NPIs are likely to vary in different host populations. For example, we found that the probability of a major outbreak depends critically on the proportion of fatal community cases that are buried unsafely when access to healthcare is limited, but when most cases are treated in healthcare facilities, we found that the extent of transmission to healthcare workers is a more important factor (electronic supplementary material, figure S3). This indicates that consideration of which NPIs to introduce should be undertaken in a bespoke, context-specific fashion. Furthermore, we found that the transmission route that contributes most to the probability of a major outbreak depends on whether the index case is treated in the community or in a healthcare facility (cf. figure 2*f* and electronic supplementary material, figure S1A), which might also be expected to vary spatially (e.g. between urban and rural locations or based on proximity to healthcare facilities).

As well as identifying key parameters underlying the probability of a major outbreak, we found in our baseline analyses that there is no single transmission route that, if eliminated, would reduce the probability of a major outbreak originating from a single community case to zero (figure 2*a*–*e*). This suggests that elimination of major Ebola outbreaks would either require NPIs that affect multiple transmission routes (e.g. community engagement and communication of the risks associated with infection may affect both the extent of community transmission and the proportion of burials that are conducted unsafely, as well as the likelihood that individuals seek treatment in healthcare facilities) or multiple NPIs to be introduced in combination. As an example, we considered the implementation of public health measures to reduce both the proportion of fatal cases in the community that are buried unsafely and the extent of transmission to healthcare workers (figure 3). We found that substantial reductions in both quantities are required to eliminate the probability of a major outbreak, which in turn requires both effective public health measures and high compliance in the host population.

Our analyses build on previous research that has sought to explore the effectiveness of NPIs in reducing EV transmission [16,39–41]. The importance of transmission at funerals in driving Ebola outbreak dynamics is well-established, with a World Health Organization report during the 2014−2016 epidemic suggesting that 80% of cases in Sierra Leone may have been linked to traditional burial and funeral practices [42]. A modelling analysis by Barbarossa *et al*. [43] involved the estimation of the basic reproduction number ($R_{0}$) in the 2014−2016 epidemic, highlighting that relaxation of control measures would lead to a substantial increase in the number of cases. Those authors identified transmission at funerals as a substantial component of the overall value of $R_{0}$. Pandey *et al.* [44] conducted a real-time analysis during the 2014−2016 epidemic, showing that multiple NPIs were required to prevent the further growth of the epidemic. Merler *et al.* [45] also unpicked the extent of transmission in different settings during that epidemic and projected the number of cases that would be averted (compared with a no interventions scenario) under different combinations of control measures. While these and other studies have quantified the level of EV transmission in a range of settings and tested different NPIs, the key novelty in the present study is that we focus on major outbreak prevention in the earliest possible stages of an outbreak through numerical calculation and analysis of the probability of a major outbreak.

Although our main focus here was the implementation of NPIs, our approach could also be used to analyse the impacts of pharmaceutical interventions on the probability of a major outbreak. While NPIs formed the cornerstone of the early response to the 2014−2016 epidemic, rapid implementation of measures such as vaccination has the potential to aid containment of future Ebola outbreaks [5,46], and ring vaccination has been adopted in recent outbreaks [47,48]. Furthermore, combinations of NPIs and pharmaceutical measures have been identified as effective strategies for countering diseases other than Ebola, such as COVID-19 [49,50] and influenza [51]. In our framework, the vaccination of at-risk communities could be modelled by reducing the values of the parameters representing the extent of transmission via different routes.

As with any modelling analysis, our study involved a range of assumptions. For example, while we used model parameter values that were based on evidence from the literature, substantial uncertainty exists in these values and transmission characteristics vary between outbreaks. For that reason, we chose to undertake global sensitivity analyses (electronic supplementary material, figures S2 and S3) as well as local sensitivity analyses (figure 2*f* and electronic supplementary material, figure S1). Additionally, we undertook a supplementary analysis in which we reproduced the results shown in figure 2, but for alternative values of our baseline model parameters. Specifically, in electronic supplementary material, figure S5, we consider a situation in which a smaller proportion of transmissions are assumed to arise at funerals. As might be expected, in that scenario, the probability of a major outbreak starting from a single community case is less sensitive to the proportion of burials that are unsafe than to some other model parameters (electronic supplementary material, figure S5F). However, our main conclusion remained unchanged: there is no single transmission route that, if eliminated, would reduce the probability of a major outbreak starting from a single community case to zero (electronic supplementary material, figure S5A–E). Reducing transmission in multiple settings is therefore crucial to eliminate the risk of major Ebola outbreaks.

It should also be noted that, in practice, factors such as the availability of specialist healthcare and the extent of unsafe burial practices would probably change during an outbreak, due to behavioural changes in the host population and the introduction of control measures (e.g. deployment of outbreak response teams [52,53]). However, since our focus was on the earliest stages of an outbreak and calculation of the probability of a major outbreak, such temporal changes when a major outbreak is ongoing would not be expected to have a substantial impact on our results. Another assumption of the transmission model underlying our analyses is that the numbers of infections arising from each case via each transmission route are assumed to be drawn from geometric distributions. While this assumption is common to many epidemiological models, other distributions could be considered. In principle, it is possible to account for superspreading when calculating the probability of a major outbreak by incorporating a negative binomial offspring distribution [21,54]. Doing so for the transmission model used here would probably come at the cost of having to estimate or assume the level of overdispersion in the number of cases occurring via each transmission route.

Despite these assumptions, in this article we were able to demonstrate how the probability of a major outbreak can be inferred using an Ebola outbreak model that accounts for multiple transmission settings. We identified key transmission routes underlying the probability of a major outbreak using local and global sensitivity analyses and showed that elimination of transmission in any individual setting would not be sufficient to prevent major Ebola outbreaks entirely. Major outbreak prevention requires coordinated public health measures that limit transmission in a range of settings, with the most effective measures depending on the characteristics of the host population within which the outbreak begins. This research provides a framework that can be used to assess the effectiveness of interventions in reducing the risk of major outbreaks for a range of infectious diseases, particularly in scenarios in which there are multiple transmission settings.

## Data Availability

The computing code used to perform the analyses in this article is available at https://github.com/abbie-evans/ebola-outbreak-risks (and archived at [55]). All code was written in Python v. 3.11.9. Supplementary material is available online [56].
